# Joint State and Fault Estimation for Nonlinear Systems Subject to Measurement Censoring and Missing Measurements

**DOI:** 10.3390/s25175396

**Published:** 2025-09-01

**Authors:** Yudong Wang, Tingting Guo, Xiaodong He, Lihong Rong, Juan Li

**Affiliations:** 1 College of Mechanical and Electrical Engineering, Qingdao Agricultural University, Qingdao 266109, China; 20232111029@stu.qau.edu.cn (Y.W.); ronglh@qau.edu.cn (L.R.); 2 School of Electromechanical and Automative Engineering, Yantai University, Yantai 264005, China; gttchn@ytu.edu.cn; 3 Achievement Transformation and Promotion Department, Shandong Academy of Agricultural Machinery Sciences, Jinan 250103, China; 18615185935@163.com

**Keywords:** joint state and fault estimation, measurement censoring, missing measurements, fusion estimation, recursive algorithms

## Abstract

This paper investigates the joint state and fault estimation problem for a class of nonlinear systems subject to both measurement censoring (MC) and random missing measurements (MMs). Recognizing that state estimation for nonlinear systems in complex environments is frequently compromised by MMs, MC phenomena, and actuator faults, a novel joint estimation framework that integrates improved Tobit Kalman filtering and federated fusion is proposed, enabling simultaneous robust estimation of system states and fault signals. Among them, the Tobit measurement model is introduced to characterize the phenomenon of MC, a set of Bernoulli random variables is used to describe the MM phenomenon and common actuator faults (abrupt and ramp faults) are considered. In the fusion estimation stage, each sensor transmits observations to the local estimator for preliminary estimation, then sends the local estimated values to the fusion center for generating fusion estimates. The local filtering error covariance is ensured and the upper bound is minimized by reasonably determining the filter gain, while the fusion center performs fusion estimation based on the federated fusion criterion. In addition, this paper proves the boundedness of the filtering error of the designed estimator under certain conditions. Finally, the effectiveness of the estimation framework is demonstrated through two engineering experiments.

## 1. Introduction

With the rapid advancement of networked systems and communication technologies, wireless sensor networks (WSNs), owing to their flexible deployment, cost-effectiveness, and low maintenance requirements, have emerged as a pivotal technology in environmental monitoring [1,2], smart control [3,4], and industrial automation [5,6]. However, WSNs still face practical challenges such as dynamic environments, communication noise, and sensor reliability. In practical engineering applications, while high-precision sensors can directly acquire state data, their prohibitive costs often render them economically unfeasible for large-scale implementations [7]. Compared with high-precision hardware sensors, soft measurement techniques offer cost-effective and flexible alternatives through algorithmic estimation. Consequently, researchers have turned to algorithm-based soft measurement techniques [8]. For instance, reference [9] demonstrated mobile robot localization in indoor environments using ceiling vision and rangefinder methods. A novel time-varying observation method was developed [10] which enables precise estimation of distinct elements in periodic load current signals, eliminating the need for additional complex processing steps. The above soft measurement techniques can all achieve the purpose of indirect measurement. However, in practical complex environments their performance depends heavily on the fusion algorithm’s robustness to noise, bias, and communication problems.

To address the above problems, considering that multi-sensor fusion estimation (MSFE) can appropriately fuse data from all sensors to obtain optimal state estimation, adopting multi-sensor collaborative work can effectively improve the robustness and accuracy of estimation [11]. Compared with using single sensor estimation, MSFE has the advantages of rich information sources, comprehensive sensing coverage, and robust performance under faulty conditions. Furthermore, MSFE can provide better estimation performance [12]. By now, MSFE has attracted extensive research attention from scholars and has achieved certain results [13,14,15]. For example, a fusion method based on SLAM cameras and inertial sensors has been designed for the precise indoor pedestrian positioning system [13]. And in reference [14], a pelvic reduction positioning and navigation method is proposed where complementary electromagnetic positioning and optical navigation systems cooperate to overcome traditional limitations, significantly enhancing positional precision while enabling both strong repositioning forces and sophisticated reduction trajectories.

In the field of industrial control, most systems exhibit nonlinear characteristics, including but not limited to satellite navigation systems [16,17,18], multi-sensor network systems [19,20,21], and power systems [22,23,24]. Such nonlinearity leads to more complex dynamic behaviors and increased susceptibility to various fault conditions [25,26]. During operation, actuators—as critical executive components of control systems—face significantly elevated risks of failure due to cumulative mechanical wear, aging electrical components, and harsh environmental influences. Notably, if faults cannot be promptly estimated/diagnosed upon occurrence, they may impair the system’s environmental perception capabilities, thereby degrading overall performance or even triggering accidents [27]. To address this challenge, researchers have developed various joint state and fault estimation (JSFE) methods for nonlinear systems, integrating state estimation with real-time fault estimation capabilities in a unified framework [27,28,29,30,31,32,33]. For example, a novel JSFE algorithm was proposed for nonlinear systems in [27] which addresses challenges arising from missing measurements, component failures, and nonlinearities. In [28], the author designed an estimator to simultaneously estimate system states and actuator efficiency loss. Despite the significant engineering value of JSFE, practical challenges still exist in applications that use low-cost sensors. These sensors, often employed in large-scale sensor networks, are prone to faults and are affected by network or environmental factors, which can lead to a significant decline in the accuracy of joint estimation.

Most current studies still rely on conventional estimation frameworks based on the Kalman filter (KF) and its several extensions. However, in practical applications, due to difficulties such as target occlusion, environmental interference, and signal loss, it is inevitable that the sensor will experience the phenomenon of measurement censoring (MC). It refers to the phenomenon where sensor data is only partially available. This problem is particularly common in WSNs, where data reliability can be compromised [34]. It makes the original filtering method difficult to apply. The common methods for state estimation problems affected by MC are to use proven nonlinear filtering techniques, such as particle filter algorithms, but they increase the computational burden accordingly. To address these problems, reference [34] proposed a novel Tobit Kalman filter (TKF) approach that not only maintains performance comparable to standard KF but also delivers accurate and unbiased estimates under partially MC conditions. Following this groundbreaking work, the state estimation/filtering problem based on TKF has aroused great interest and achieved some notable advancements, for instance, an innovative local TKF designed for networked systems subject to MC and measurement outliers [35]. Reference [30] developed a dynamically event-triggered TKF for nonlinear systems with parameter uncertainty and MC, establishing mean-square stability conditions for the JSFE problem. At present, most research still focuses on linear systems, which is obviously inconsistent with the nonlinear characteristics in practical engineering. Dynamic systems such as robot motion, power systems, chemical processes, etc., often exhibit complex behaviors such as strong nonlinearity and time-varying parameters. Therefore, improving the existing TKF fusion algorithm for nonlinear systems has become a critical challenge requiring immediate attention in state estimation research.

Beyond the above difficulties, sensor data may suffer from network-induced phenomena during acquisition and transmission due to environmental factors and limited network bandwidth. These phenomena can compromise the stability and reliability of data transmission, severely degrade estimation performance and potentially destabilize the controlled system. Common network-induced phenomena include time delays [36,37], signal quantization [38,39], channel fading [40,41], and missing measurements (MMs) [42,43]. Among these, MMs refer to data loss at certain time instants due to various factors; it can prevent the estimator from obtaining real-time state information. When measurement deficiencies occur, the system may fail to accurately obtain status feedback information, leading to the failure of control strategies or performance degradation. If the actuator also malfunctions at this time, the controllability of the system will further deteriorate and may even lead to catastrophic consequences. It is noteworthy that MMs frequently occur in complex nonlinear systems. Recent studies have begun addressing JSFE under MMs [27,42,43,44,45,46]. In reference [42], the authors developed a state estimator for power distribution systems with MMs using linear matrix inequality techniques and matrix theory, with simulations demonstrating its robustness and anti-disturbance capability against random MMs. And in reference [46], the fault detection problem under MMs was first investigated for nonlinear systems.

So far, although there have been relevant research results on estimation in the event of MMs, there is still a lack of comprehensive solutions for problems such as MC, MMs, and actuator faults. Thus, the purpose of this paper is to propose a JSFE algorithm based on multi-sensor fusion to address the above-mentioned problems. Compared with the existing methods, the difficulty of this paper lies in the following questions. (1) What modeling approach can effectively capture the interdependent impacts of MC, MMs, and actuator faults in a unified regression framework? (2) How can the tight couplings of the MMs, the covariance bounds as well as the filter gains be handled? (3) How can the error covariance boundedness to validate the proposed fusion estimator’s performance guarantees be established?

The main contributions are threefold. (1) A new Tobit model has been developed to handle the JSFE problem with MC, MMs, and actuator faults simultaneously; (2) a novel JSFE algorithm is designed, and the upper bound (UB) of a set of local filtering error covariance (FEC) is obtained with the designed local and fusion estimators to minimize the UB; (3) boundedness analysis of the JSFE algorithm is established under specified conditions to evaluate its performance characteristics, and the correctness of the conclusion is verified by examples.

The remainder of this paper is organized as follows. Section 2 presents the modeling of nonlinear systems incorporating MC, MMs, and actuator faults. Section 3 proposes a novel fusion algorithm for JSFE in nonlinear systems. Section 4 empirically validates the effectiveness of the proposed algorithm through two representative engineering case studies. Section 5 concludes the paper with relevant findings.

**Notation** **1.** *$R^{n}$ denotes the n-dimensional Euclidean space. $\mathrm{Pr}\;\left\{\beta =1\right\}=\bar{\beta }$ means the probability of the stochastic variable β=1 occurring is $\bar{\beta }$. T” denotes the transpose of a matrix. diag{•} indicates a diagonal matrix. $E\left\{\beta \right\}$ expresses the expectation of β. Other notations used are quite standard.*

## 2. System Modeling

**Figure 1:**
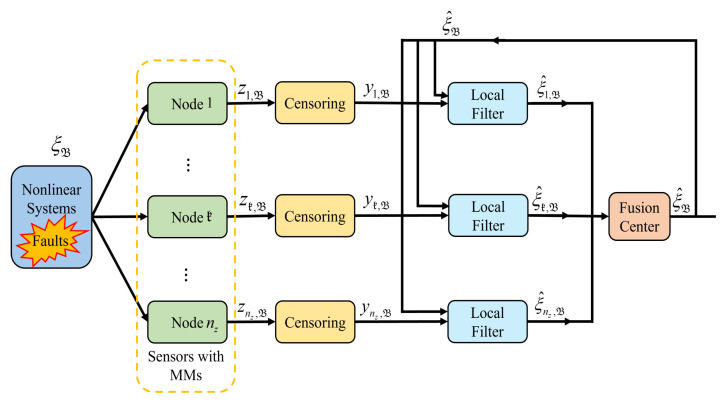
Schematic of the considered problem.

As illustrated in Figure 1, the system state is subject to random actuator faults. MMs are prone to occur when local sensors are used for measurement. The transmitted measurements may undergo censoring upon arrival at the filter. Ultimately, the estimated values of local nodes are fused at the fusion center. The following section introduces the random MC, MMs, actuator faults, and federated fusion criterion in a rigorous mathematical manner.

### 2.1. System Description

Consider the following nonlinear systems subject to actuator faults [47]:

(1) $${\vec{\xi }}_{B+1}={\vec{A}}_{B}{\vec{\xi }}_{B}+{\vec{F}}_{B}f_{B}+G\left({\vec{\xi }}_{B}\right)+{\vec{B}}_{B}{\vec{\omega }}_{B},$$

where ${\vec{\xi }}_{B}\in R^{n_{\xi }}$ is the state vector; ${\vec{A}}_{B}$, ${\vec{F}}_{B}$ and ${\vec{B}}_{B}$ are known matrices; ${\vec{\omega }}_{B}$ is the process noise; and $f_{B}$ is the fault signal.

The nonlinear function $G({\vec{\xi }}_{B})$ with G(0)=0 satisfies the Lipschitz condition:

(2) $$∥G(ı)-G(ȷ)∥\leq ∥G_{B}\left(\iota -ȷ\right)∥,$$

where $G_{B}$ is a known matrix.

Denote $f_{B}^{\left[0\right]}≜f_{B}$ and $f_{B}^{\left[0\right]}≜f_{B+1}^{\left[0-1\right]}-f_{B}^{\left[0\right]}$ (B≥1) with $f_{0}=0$. In this way, this formulation accommodates a broad spectrum of faults [48]. Consider the case where the temporal evolution and dynamic characteristics of actuator faults are not known in advance. The random bias ${\vec{\omega }}_{B}^{f}$ first-order random bias of the validity factor with a large covariance is considered, i.e.,

(3) $$f_{B+1}^{\left[1\right]}≜f_{B}^{\left[1\right]}+{\vec{\omega }}_{B}^{f},$$

where ${\vec{\omega }}_{B}^{f}$ is the fault noise with covariance ${\vec{Q}}_{B}^{f}$.

**Remark** **1.** *Real-world applications face inherent difficulties in handling faults with entirely unknown dynamics. Therefore, for both abrupt and ramp faults, the adopted assumption is that fault increments remain nearly constant, subject only to Gaussian noise disturbances.*

The random-bias representation is employed due to its ability to model various fault types, such as abrupt and ramp faults. This holds true under the condition that the fault signal’s second-order difference equals zero [49].

### 2.2. Measurement Model

Its corresponding output measurement is

(4) $$z_{k,B}=D_{k,B}{\vec{C}}_{k,B}{\vec{\xi }}_{B}+v_{k,B},\;k=1,2,\ldots ,p,$$

where $z_{k,B}\in R^{n_{z}}$ is the observation vector; *p* is the number of sensors; ${\vec{C}}_{k,B}$ is a known matrix; and $v_{k,B}$ is the measurement noise. Define a set of mutually independent Bernoulli distribution variables $D_{k,B}$ to describe a random occurrence of MMs. It is assumed that $E\left\{D_{k,B}\right\}={\bar{D}}_{k,B}$. Denote the measurements collected by node k as:

$$z_{k,B}≜{\left\{z_{k,B}^{1},z_{k,B}^{2},\ldots ,z_{k,B}^{m}\right\}}^{T},$$

where $k\in N_{k}$ and $z_{k,B}^{m}$ ($m\in \{1,2,\ldots ,n_{z}\}$) is the *m*-th entry of $z_{k,B}$.

### 2.3. Augmented Matrix

Let $\xi_{B}≜{\left[{\vec{\xi }}_{B}^{T}\;f_{B}\;f_{B}^{\left[1\right]}\right]}^{T}$. From (1) and (4), the following augmentation system is derived:

(5) $$\left\{\begin{array}{c} \xi_{B+1}=A_{B}\xi_{B}+G\left(\xi_{B}\right)+B_{B}\omega_{B},\\ z_{k,B}=D_{k,B}C_{k,B}\xi_{B}+v_{k,B}, \end{array}\right.$$

where

$$A_{B}=\left[\begin{array}{ccc} {\vec{A}}_{B} & {\vec{F}}_{B} & 0\\ 0 & I & I\\ 0 & 0 & I \end{array}\right],\;G\left(\xi_{B}\right)=\left[\begin{array}{c} G({\vec{\xi }}_{B})\\ 0\\ 0 \end{array}\right],$$

$$B_{B}=\left[\begin{array}{ccc} {\vec{B}}_{B} & 0 & 0\\ 0 & 0 & 0\\ 0 & 0 & I \end{array}\right],\;\omega_{B}=\left[\begin{array}{c} {\vec{\omega }}_{B}\\ 0\\ {\vec{\omega }}_{B}^{f} \end{array}\right],$$

$$v_{k,B}={\vec{v}}_{k,B},\;C_{k,B}=\left[\;{\vec{C}}_{k,B}\;0\;0\right].$$

### 2.4. Measurement Censoring Model

The MC phenomenon arises when sensor outputs change continuously with system states inside a specific dynamic range but stay constant beyond it. Near the censoring boundaries, measurement noise becomes non-Gaussian with completely unknown statistics. The MC status of $y_{k,B}$ is determined by the Tobit model as:

(6) $$y_{k,B}^{m}=\left\{\begin{array}{cc} z_{k,B}^{m}, & z_{k,B}^{m}>\tau_{k}^{m},\\ \tau_{k}^{m}, & z_{k,B}^{m}\leq \tau_{k}^{m}, \end{array}\right.$$

where $y_{k,B}^{m}$ represents the physically measured value acquired from node k, which is subsequently compared against the predetermined threshold value $\tau_{k}^{m}$ for censoring determination.

Consider the model (6), then define the occurrence of MC in $y_{k,B}^{m}$ through a binary random variable $\gamma_{k,B}^{m}$ that follows a Bernoulli distribution:

(7) $$\gamma_{k,B}^{m}=\left\{\begin{array}{cc} 1, & z_{k,B}^{m}>\tau_{k}^{m},\\ 0, & z_{k,B}^{m}\leq \tau_{k}^{m}, \end{array}\right.$$

and the probability distribution is as follows:

(8) $$\left\{\begin{array}{c} \mathrm{Pr}\;\left\{\gamma_{k,B}^{m}=1\right\}=\gamma_{k,B}^{m},\\ \mathrm{Pr}\;\left\{\gamma_{k,B}^{m}=0\right\}=1-\gamma_{k,B}^{m}. \end{array}\right.$$

Furthermore, based on (7), $y_{k,B}$ can be rewritten as follows:

(9) $$y_{k,B}^{m}=\gamma_{k,B}^{m}z_{k,B}^{m}+\left(1-\gamma_{k,B}^{m}\right)\tau_{k}^{m}.$$

The random variable $\gamma_{k,B}$ is specifically designed to characterize the MC in measurement $z_{k,B}$. Motivated by [34] and (5), the approximate value of ${\bar{\gamma }}_{k,B}^{m}$ is

(10) $${\bar{\gamma }}_{k,B}^{m}=\Phi \;\left(\frac{{\bar{D}}_{k,B}C_{k,B}^{m}{\hat{\xi }}_{B}^{-,m}-\tau_{k}^{m}}{\sqrt{R_{k,B}^{m,m}}}\right),$$

where ${\hat{\xi }}_{B}^{-,m}$ is the *m*th estimate of $\xi_{B}^{m}$; $C_{k,B}^{m}$ is the *m*th row vector of matrix $C_{k,B}$; and Φ(·) is the CDF of standard normal distributed variables.

Denote

$$\begin{array}{cc} y_{k,B} & ≜{\left\{y_{k,B}^{1},y_{k,B}^{2},\ldots ,y_{k,B}^{n_{z}}\right\}}^{T},\\ L_{k,B} & ≜\mathrm{diag}\{\gamma_{k,B}^{1},\gamma_{k,B}^{2},\ldots ,\gamma_{k,B}^{n_{z}}\},\\ {\bar{L}}_{k,B} & ≜\mathrm{diag}\{{\bar{\gamma }}_{k,B}^{1},{\bar{\gamma }}_{k,B}^{2},\ldots ,{\bar{\gamma }}_{k,B}^{n_{z}}\},\\ \;C_{k} & ≜\mathrm{diag}\{\tau_{k}^{1},\tau_{k}^{2},\ldots ,\tau_{k}^{n_{z}}\}. \end{array}$$

Consequently, measurement model (9) is augmented as follows:

(11) $$y_{k,B}=L_{k,B}z_{k,B}+\left(1-L_{k,B}\right)C_{k}.$$

**Assumption** **1.** *The initial state vector $\xi_{k,0}$ follows a Gaussian distribution with mean ${\bar{\xi }}_{k,0}$ and covariance matrix $P_{k,0}$. The noises ${\vec{\omega }}_{B}$ and $v_{k,B}$ constitute mutually independent, zero-mean white Gaussian sequences characterized by covariance matrices ${\vec{Q}}_{B}$ and $R_{k,B}$, respectively. Furthermore, $\xi_{k,0}$, ${\vec{\omega }}_{B}$, ${\vec{\omega }}_{B}^{f}$, $\gamma_{k,B}^{m}$, $v_{k,B}$ and $D_{k,B}$ are uncorrelated.*

This research is concerned with designing fusion estimators for the following structures based on dynamical systems (5) and measurement models (11):

(12) $$\left\{\begin{array}{cc} {\hat{\xi }}_{B} & =\sum_{k=1}^{p}G_{k,B}{\hat{\xi }}_{k,B},\\ {\hat{\xi }}_{k,B}^{-} & =A_{B-1}{\hat{\xi }}_{B-1}+G\left({\hat{\xi }}_{B-1}\right),\\ {\hat{\xi }}_{k,B} & ={\hat{\xi }}_{k,B}^{-}+K_{k,B}\left(y_{k,B}-{\hat{y}}_{k,B}^{-}\right), \end{array}\right.$$

where ${\hat{\xi }}_{k,B}$ and ${\hat{\xi }}_{k,B}^{-}$ are the estimate and prediction of state $\xi_{B}$ from *note*k. The matrix weights $G_{k,B}$ and the local filter gains $K_{k,B}$ will be determined later.

Let ${\tilde{\xi }}_{k,B}≜\xi_{B}-{\hat{\xi }}_{k,B}$ and ${\tilde{\xi }}_{k,B}^{-}≜\xi_{B}-{\hat{\xi }}_{k,B}^{-}$ be the local filtering and local prediction error. Then define $P_{k,B}≜E\;\left\{{\tilde{\xi }}_{k,B}{\tilde{\xi }}_{k,B}^{T}\right\},$ and $P_{k,B}^{-}≜E\;\left\{{\tilde{\xi }}_{k,B}^{-}{\left({\tilde{\xi }}_{k,B}^{-}\right)}^{T}\right\}$ to be the local FEC and the local prediction error covariance (PEC).

This paper aims to develop a multi-sensor fusion estimator for JSFE with three design objectives: (1) the UB is guaranteed on local FECs; (2) the attained UBs are minimized through determining appropriate local filter gains; (3) estimation performance is rigorously evaluated through boundedness analysis of the minimized error covariances.

## 3. Main Result

A novel JSFE algorithm is developed by employing matrix theory and mathematical induction techniques to derive the exact filter gain formulation in this section. Then, some key lemmas are presented to support the subsequent theoretical derivation.

**Lemma** **1** ([30])**.** *For arbitrary vectors Q and P, the following inequality relations are satisfied:*

(13) $$QP^{T}+PQ^{T}\leq bQQ^{T}+b^{-1}PP^{T},$$

*where b is a positive scalar quantity.*

**Lemma** **2** ([50])**.** *If the sequence matrix functions $\varpi \left(\cdot \right):R^{n}\;\to \;R^{n}$, $J\left(\cdot \right):R^{n}\;\to \;R^{n}$, 0≤B≤N satisfy *

(14) $$\begin{array}{cc} \varpi_{B}\left(M\right)=\varpi_{B}^{T}\left(M\right), & \forall \;M=M^{T}>0,\\ J_{B}\left(M\right)=J_{B}^{T}\left(M\right), & \end{array}$$

*and*

(15) $$\begin{array}{cc} \varpi_{B}\left(M\right)\leq \varpi_{B}\left(N\right), & \forall \;M=M^{T}\leq N=N^{T},\\ \varpi_{B}\left(N\right)\leq J_{B}\left(N\right), & \end{array}$$

*then, if the matrix functions $M_{B}$ and $N_{B}$ satisfy*

(16) $$M_{B+1}=\varpi_{B}\left(M_{B}\right),\;N_{B+1}=\varpi_{B}\left(N_{B}\right),\;M_{0}=N_{0}>0,$$

*one has $M_{B}\leq N_{B}$.*

**Lemma** **3.** *According to [51] and* (5), *the mathematical expectation and covariance of $y_{k,B}^{m}$ are*

(17) $$\begin{array}{cc} E\{y_{k,B}^{m}|\xi_{B}\} & =\left[1-\Phi \;\left(\chi_{k,B}^{m}\right)\right]\left[{\bar{D}}_{k,B}^{m}C_{k,B}^{m}{\hat{\xi }}_{k,B}^{-,m}+\sqrt{R_{k,B}^{m,m}}\;\lambda \;\left(\chi_{k,B}^{m}\right)\right]+\Phi \;\left(\chi_{k,B}^{m}\right)\;\tau_{k}^{m}, \end{array}$$

(18) $$\mathrm{var}\{y_{k,B}^{m}|\xi_{B}\}=\sqrt{R_{k,B}^{m,m}}\left[1+\varphi \;\left(\chi_{k,B}^{m}\right)\right],$$

*where*

$$\begin{array}{cc} & \chi_{k,B}^{m}≜\frac{\tau_{k}^{m}-{\bar{D}}_{B}C_{k,B}^{m}{\hat{\xi }}_{k,B}^{-,m}}{\sqrt{R_{k,B}^{m,m}}},\\ & \varphi (\cdot )=\lambda (\cdot )\;[\lambda (\cdot )-(\cdot )],\;\\ & \lambda \left(\cdot \right)=\frac{\phi (\cdot )}{1-\Phi (\cdot )}, \end{array}$$

*and ϕ(·) is the PDF of the random variable “·”.*

Denote

$$\begin{array}{cc} \mathrm{var}\;\{\;y_{k,B}\;\left|\;\xi_{B}\right\} & ≜{\left[\mathrm{var}\;\left\{y_{k,B}^{1}\;|\;\xi_{B}\right\},\mathrm{var}\;\left\{y_{k,B}^{2}\;|\;\xi_{B}\right\},\ldots ,\mathrm{var}\;\left\{y_{k,B}^{n_{z}}\;|\;\xi_{B}\right\}\right]}^{T},\\ {\hat{y}}_{k,B}^{-} & ≜{\left[E\;\left\{y_{k,B}^{1}\;|\;\xi_{B}\right\},E\;\left\{y_{k,B}^{2}\;|\;\xi_{B}\right\},\ldots ,E\;\left\{y_{k,B}^{n_{z}}\;|\;\xi_{B}\right\}\right]}^{T},\\ \lambda \;\left(\chi_{k,B}\right) & ≜\mathrm{diag}\;\left\{\lambda \left(\chi_{k,B}^{1}\right),\lambda \left(\chi_{k,B}^{2}\right),\ldots ,\lambda \left(\chi_{k,B}^{n_{z}}\right)\right\},\\ \varphi \;\left(\chi_{k,B}\right) & ≜\mathrm{diag}\;\left\{\varphi \left(\chi_{k,B}^{1}\right),\varphi \left(\chi_{k,B}^{2}\right),\ldots ,\varphi \left(\chi_{k,B}^{n_{z}}\right)\right\}. \end{array}$$

Consequently, (17) and (18) are augmented as follows:

(19) $$\begin{array}{cc} \mathrm{var}\{\left.y_{k,B}\right|\xi_{B}\}= & \sqrt{R_{k,B}}\left[1+\varphi \left(\chi_{k,B}\right)\right],\\ {\hat{y}}_{k,B}^{-}= & \left[1-\;\;\;\Phi \;\;\;\left(\chi_{k,B}\right)\right]\left[{\bar{D}}_{k,B}C_{k,B}{\hat{\xi }}_{k,B}^{-}\right.+\sqrt{R_{k,B}}\left.\;\;\;\lambda \;\;\;\left(\chi_{k,B}\right)\right]\\ \; & \;\;+\;\;\;\Phi \;\;\;\left(\chi_{k,B}\right)C_{k}, \end{array}$$

**Lemma** **4.** *The PEC and FEC of the local estimators are given by*

(20) $$\begin{array}{cc} P_{k,B}^{-}= & \;A_{B}P_{k,B}A_{B}^{T}+B_{B}Q_{B}B_{B}^{T}+E\{\left[G\left(\xi_{B}\right)-G\left({\hat{\xi }}_{k,B}\right)\right]\\ & \;\times \left[G\left(\xi_{B}\right)-G\left({\hat{\xi }}_{k,B}\right){]}^{T}\right\}+A_{B}+A_{B}^{T}, \end{array}$$

(21) $$\begin{array}{cc} P_{k,B}= & \;\left(I-K_{k,B}{\bar{L}}_{k,B}{\bar{D}}_{k,B}C_{k,B}\right)P_{k,B}^{-}(I-K_{k,B}{\bar{L}}_{k,B}{\bar{D}}_{k,B}\\ & \;\times C_{k,B}{)}^{T}+K_{k,B}E\left\{V_{k,B}V_{k,B}^{T}\right\}K_{k,B}^{T}+B_{k,B}+B_{k,B}^{T}, \end{array}$$

*where*

$$\begin{array}{cc} V_{k,B}= & {\bar{L}}_{k,B}\left(D_{k,B}-{\bar{D}}_{k,B}\right)C_{k,B}\xi_{B}+\left(L_{k,B}-{\bar{L}}_{k,B}\right)D_{k,B}C_{k,B}\xi_{B}\\ & \;\;+L_{k,B}v_{k,B}+\left({\bar{L}}_{k,B}-L_{k,B}\right)C_{k}-{\bar{L}}_{k,B}\sqrt{R_{k,B}}\;\;\;\lambda \;\;\;\left(\chi_{k,B}\right),\\ B_{k,B}= & E\left\{\left(I-K_{k,B}{\bar{L}}_{k,B}{\bar{D}}_{k,B}C_{k,B}\right){\tilde{\xi }}_{k,B}^{-}V_{k,B}K_{k,B}^{T}\right\},\\ A_{B}= & A_{B}E\left\{{\tilde{\xi }}_{B}{\left[G\left(\xi_{B}\right)-G\left({\hat{\xi }}_{k,B}\right)\right]}^{T}\right\}. \end{array}$$

**Proof.**  Remembering the definition of ${\hat{\xi }}_{k,B}^{-}$, and subtracting (12) from (5), one can obtain the following:

(22) $$\begin{array}{cc} \; & {\tilde{\xi }}_{k,B+1}^{-}=\xi_{B+1}-{\hat{\xi }}_{k,B+1}^{-}\\ \; & \;=A_{B}{\tilde{\xi }}_{k,B}+G\left(\xi_{B}\right)-G\left({\hat{\xi }}_{k,B}\right)+B_{B}\omega_{B}. \end{array}$$

The expectation of the measurement $y_{k,B}$ is defined as ${\hat{y}}_{k,B}^{-}≜E\left\{\left.y_{k,B}\right|y_{1:k,B-1}\right\}$, which is conditioned on the measurement sequence $y_{1:k,B-1}$. Based on (5) and (12), the FEC is derived as:

(23) $${\tilde{\xi }}_{k,B}={\tilde{\xi }}_{k,B}^{-}-K_{k,B}\left(y_{k,B}-{\hat{y}}_{k,B}^{-}\right).$$

Denote ${\tilde{y}}_{k,B}^{-}≜y_{k,B}-{\hat{y}}_{k,B}^{-}$, and integrate (11) and (19), then it is not hard to get

(24) $$\begin{array}{cc} {\tilde{y}}_{k,B}^{-}= & L_{k,B}D_{k,B}\left(C_{k,B}\xi_{B}+v_{k,B}\right)+\left(I-L_{k,B}\right)C_{k}-{\bar{L}}_{k,B}[{\bar{D}}_{k,B}C_{k,B}{\hat{\xi }}_{k,B}^{-}\\ \; & \;+\sqrt{R_{k,B}}\lambda \left(\chi_{k,B}\right)]+\left(I-{\bar{L}}_{k,B}\right)C_{k},\\ \;= & {\bar{L}}_{k,B}{\bar{D}}_{k,B}C_{k,B}{\tilde{\xi }}_{k,B}^{-}+{\bar{L}}_{k,B}\left(D_{k,B}-{\bar{D}}_{k,B}\right)\xi_{B}+\left(L_{k,B}-{\bar{L}}_{k,B}\right)D_{k,B}\xi_{B}\\ \; & \;+L_{k,B}v_{k,B}+\left({\bar{L}}_{k,B}-L_{k,B}\right)C_{k}-{\bar{L}}_{k,B}\sqrt{R_{k,B}}\;\;\;\lambda \;\;\;\left(\chi_{k,B}\right). \end{array}$$

Combining (22)–(24), the local one-step PECs and the FECs which are shown in (20) and (21) can be obtained. By now, the derivation has been completed.    □

The results of Lemma 4 confirm the boundedness and feasibility of the intermediate estimation variables. Now, the first main theoretical guarantee will be established. Theorem 1 builds directly upon Lemma 4 to obtain the UB in explicit terms through a recursive relationship established by (19) and (20). Meanwhile, we present a parameterization of the local estimator gain $K_{k,B}$ to obtain the results by minimizing the UB derived.

**Theorem** **1.** *Suppose that positive scalars $b_{k}\left(k=1,2,3\right)$ and the initial condition $\sum_{k,0}=P_{k,0}$ are given. If there is a matrix sequence $\sum_{k,B}$ satisfying the following:*

(25) $$\begin{array}{cc} \sum_{k,B}= & \left(1+b_{2}\right)\left(I-K_{k,B}{\bar{L}}_{k,B}{\bar{D}}_{k,B}C_{k,B}\right)\sum_{k,B}^{-}(I-K_{k,B}{\bar{L}}_{k,B}{\bar{D}}_{k,B}\\ & \;\times C_{k,B}{)}^{T}+\left(1+b_{2}^{-1}\right)\left(1+b_{3}\right)K_{k,B}\mathrm{var}\left\{\left.y_{k,B}\right|\xi_{B}\right\}K_{k,B}^{T}+(1\\ & \;+b_{2}^{-1})\left(1+b_{3}^{-1}\right)K_{k,B}{\bar{L}}_{k,B}{\bar{D}}_{k,B}\sum_{k,B}^{-}{\left(K_{k,B}{\bar{L}}_{k,B}{\bar{D}}_{k,B}C_{k,B}\right)}^{T}, \end{array}$$

*where*

$$\begin{array}{cc} \sum_{k,B}^{-}= & \left(1+b_{1}\right)A_{B-1}\sum_{k,B-1}A_{B-1}^{T}+\left(1+b_{1}^{-1}\right)G_{B-1}\sum_{k,B-1}G_{B-1}^{T}\\ & +B_{B-1}Q_{B-1}B_{B-1}^{T}, \end{array}$$

*then the UB on the $\sum_{k,B}$ can be minimized by $K_{k,B}$, designated as:*

(26) $$\begin{array}{cc} K_{k,B}= & \left(1+b_{2}\right)\sum_{k,B}^{-}{\left({\bar{L}}_{k,B}{\bar{D}}_{k,B}C_{k,B}\right)}^{T}(\left(1+b_{2}\right)\left({\bar{L}}_{k,B}{\bar{D}}_{k,B}C_{k,B}\right)\\ & \;\times \sum_{k,B}^{-}{\left({\bar{L}}_{k,B}{\bar{D}}_{k,B}C_{k,B}\right)}^{T}+\left(1+b_{2}^{-1}\right)\left(1+b_{3}\right)\mathrm{var}\left\{\left.y_{k,B}\right|\xi_{B}\right\}\\ & \;+\left(1+b_{2}^{-1}\right)\left(1+b_{3}^{-1}\right){\bar{L}}_{k,B}{\bar{D}}_{k,B}C_{k,B}\sum_{k,B}^{-}{\left({\bar{L}}_{k,B}{\bar{D}}_{k,B}C_{k,B}\right)}^{T}{)}^{-1}. \end{array}$$

*Furthermore, the minimum UB $\sum_{k,B}^{\min}$ is calculated by*

(27) $$\sum_{k,B}^{\min}=\left(1+b_{2}\right)\left(I-K_{k,B}{\bar{L}}_{k,B}{\bar{D}}_{k,B}C_{k,B}\right)\sum_{k,B}^{-,\min}.$$

**Proof.**  It can be deduced from (3):

(28) $$E\left\{\left[G\left(\xi_{B}\right)-G\left({\hat{\xi }}_{k,B}\right)\right]{\left[G\left(\xi_{B}\right)-G\left({\hat{\xi }}_{k,B}\right)\right]}^{T}\right\}\leq G_{B}P_{k,B}G_{B}^{T}.$$

Apply Lemma 1, then it can be deduced that

(29) $$\begin{array}{cc} A_{B}+A_{B}^{T}\;\leq & b_{1}A_{B}P_{k,B}A_{B}^{T}+b_{1}^{-1}G_{B}P_{k,B}G_{B}^{T},\\ B_{k,B}+B_{k,B}^{T}\leq & b_{2}\left(I-K_{k,B}{\bar{L}}_{k,B}{\bar{D}}_{k,B}C_{k,B}\right)P_{k,B}^{-}(I-K_{k,B}{\bar{L}}_{k,B}{\bar{D}}_{k,B}\\ \; & \;\times C_{k,B}{)}^{T}{+b}_{2}^{-1}K_{k,B}E\left\{V_{k,B}V_{k,B}^{T}\right\}K_{k,B}^{T}. \end{array}$$

According to (20) and (29), it can be obtained that:

(30) $$P_{k,B+1}^{-}\leq \left(1+b_{1}\right)A_{B}P_{k,B}A_{B}^{T}+\left(1+b_{1}^{-1}\right)G_{B}P_{k,B}G_{B}^{T}+B_{B}Q_{B}B_{B}^{T}.$$

Combining (21), (28)–(30), it can be concluded that:

(31) $$\begin{array}{cc} P_{k,B}\leq & \left(1+b_{2}\right)\left(I-K_{k,B}{\bar{L}}_{k,B}{\bar{D}}_{k,B}C_{k,B}\right)P_{k,B}^{-}(I-K_{k,B}{\bar{L}}_{k,B}\\ \; & \;\times {\bar{D}}_{k,B}C_{k,B}{)}^{T}+\left(1+b_{2}^{-1}\right)K_{k,B}E\left\{V_{k,B}V_{k,B}^{T}\right\}K_{k,B}^{T}. \end{array}$$

Remember the definition of ${\tilde{y}}_{k,B}^{-}$, then one can derive the following formula from (23) and (24):

(32) $${\tilde{y}}_{k,B}^{-}={\bar{L}}_{k,B}{\bar{D}}_{k,B}C_{k,B}{\tilde{\xi }}_{k,B}^{-}+V_{k,B},$$

then one can get

(33) $$\begin{array}{cc} E\{V_{k,B}V_{k,B}^{T}\}= & E\{\left({\tilde{y}}_{k,B}^{-}-{\bar{L}}_{k,B}{\bar{D}}_{k,B}C_{k,B}{\tilde{\xi }}_{k,B}^{-}\right)({\tilde{y}}_{k,B}^{-}{\bar{L}}_{B}\\ \; & \;-{\bar{L}}_{k,B}{\bar{D}}_{k,B}C_{k,B}{\tilde{\xi }}_{k,B}^{-}{)}^{T}\}\\ \;= & \mathrm{var}\left\{\left.y_{k,B}\right|\xi_{B}\right\}-E\left\{{\tilde{y}}_{k,B}^{-}{\left({\bar{L}}_{k,B}{\bar{D}}_{k,B}C_{k,B}{\tilde{\xi }}_{k,B}^{-}\right)}^{T}\right\}\\ \; & \;-E\left\{{\bar{L}}_{k,B}{\bar{D}}_{k,B}C_{k,B}{\tilde{\xi }}_{k,B}^{-}{\left({\tilde{y}}_{k,B}^{-}\right)}^{T}\right\}+{\bar{L}}_{k,B}{\bar{D}}_{k,B}\\ \; & \;\times C_{k,B}P_{k,B}^{-}{\left({\bar{L}}_{k,B}{\bar{D}}_{k,B}C_{k,B}\right)}^{T}. \end{array}$$

Using Lemma 1, it is not hard to get

(34) $$\begin{array}{cc} E\{V_{k,B}V_{k,B}^{T}\}\leq & \left(1+b_{3}\right)\mathrm{var}\left\{\left.y_{k,B}\right|\xi_{B}\right\}+\left(1+b_{3}^{-1}\right){\bar{L}}_{k,B}{\bar{D}}_{k,B}C_{k,B}\\ \; & \;\times P_{k,B}^{-}\;{\left({\bar{L}}_{k,B}{\bar{D}}_{k,B}C_{k,B}\right)}^{T}. \end{array}$$

Combining (30), (31), (34) and using Lemma 2, it can be deduced that:

(35) $$P_{k,B}\leq \sum_{k,B},$$

for all B≥0, i.e., $\sum_{k,B}$ is the UB of $P_{k,B}$.

The optimal gain matrix $K_{k,B}$ can be obtained by setting the partial derivative of $\sum_{k,B}$ with respect to $K_{k,B}$ as follows:

(36) $$\begin{array}{cc} \mathrm{tr}(\sum_{k,B})= & \mathrm{tr}(\left(1+b_{2}\right)\left(I-K_{k,B}{\bar{L}}_{k,B}{\bar{D}}_{k,B}C_{k,B}\right)\sum_{k,B}^{-}(I-K_{k,B}{\bar{L}}_{k,B}\\ \; & \;\times {\bar{D}}_{k,B}C_{k,B}{)}^{T}+\left(1+b_{2}^{-1}\right)\left(1+b_{3}\right)K_{k,B}\mathrm{var}\left\{\left.y_{k,B}\right|\xi_{k,B}\right\}\\ \; & \;\times K_{k,B}^{T}+\left(1+b_{2}^{-1}\right)\left(1+b_{3}^{-1}\right)K_{k,B}{\bar{L}}_{k,B}{\bar{D}}_{k,B}C_{k,B}\sum_{k,B}^{-}\\ \; & \;\times {\left(K_{k,B}{\bar{L}}_{k,B}{\bar{D}}_{k,B}C_{k,B}\right)}^{T}). \end{array}$$

Let $\frac{\partial (\mathrm{tr}(\sum_{k,B}))}{\partial (K_{k,B})}=0$, then the formula (26) can be obtained. Substituting (26) into (25), the minimized UB $\sum_{k,B}^{\min}$ is shown in (27). The proof is now complete.    □

With the minimized UB $\sum_{k,B}^{\min}$ in hand, the weight matrix $G_{k,B}$ can be computed following the federated fusion rule as:

(37) $$G_{k,B}=\sum_{B}^{\min}{\left(\sum_{k,B}^{\min}\right)}^{-1},$$

where $\sum_{B}^{\min}$ corresponds to the minimal fused upper bound, given as:

(38) $$\sum_{B}^{\min}={\left({\sum_{k=1}^{p}\left(\sum_{k,B}^{\min}\right)}^{-1}\right)}^{-1}.$$

At this stage, the design process for the fusion estimator (12) has been completed. A critical implementation aspect requires following the federated fusion rule’s information sharing mechanism. Therefore, initialization values for the local estimate, UB, and noise covariance at time B−1 can be set as:

(39) $$\left\{\begin{array}{cc} \;\;{\hat{\xi }}_{k,B-1} & ≜{\hat{\xi }}_{B-1},\\ \;\sum_{k,B-1}^{\min} & ≜A_{k}^{-1}\sum_{B-1}^{\min},\\ Q_{k,B-1} & ≜A_{k}^{-1}\;Q_{B-1}, \end{array}\right.$$

where $\sum_{k=1}^{p}A_{k}≜1$.

The next objective is to formulate sufficient conditions that prove the filtering error’s boundedness in mean-square terms in the theorem below.

**Assumption** **2.** *There exist positive real numbers $\bar{a},\bar{b},\bar{c},\bar{e},\bar{g},\bar{k},\bar{q},\bar{\mu },\underset{-}{\theta }$ and $\bar{\theta }$ such that the following matrices are bound, that is*

(40) $$\begin{array}{c} ∥A_{k,B}∥\leq \bar{a},\;∥B_{k,B}∥\leq \bar{b},\;\underset{-}{c}\leq ∥C_{k,B}∥\leq \bar{c},\\ ∥Q_{k,B}∥\leq \bar{q},\;∥G_{k,B}∥\leq \bar{g}\;,\;\underset{-}{\theta }\leq ∥L_{k,B}∥\leq \bar{\theta },\\ \;\underset{-}{e}\leq ∥{\bar{D}}_{k,B}∥\leq \bar{e},\;∥\mathrm{var}\{\left.y_{k,B}\right|\xi_{k,B}\}∥\leq \bar{\mu }. \end{array}$$

**Theorem** **2.** *Consider* (1) *and* (12). *And under the premise of Assumption 2, if there is an inequality that satisfies*

(41) $$\left(\left(1+b_{2}\right){\bar{\kappa }}^{2}+\left(1+b_{2}^{-1}\right)\left(1+b_{3}^{-1}\right){\bar{k}}^{2}{\bar{\theta }}^{2}{\bar{c}}^{2}\right)\left(\left(1+b_{1}\right){\bar{a}}^{2}+\left(1+b_{1}^{-1}\right){\bar{g}}^{2}\right)<1,$$

*then the developed filter guarantees the mean-square bounded estimation error, expressed mathematically as:*

(42) $$\underset{B\in N}{\sup}\;E\left\{{\tilde{\xi }}_{B+1}{\tilde{\xi }}_{B+1}^{T}\right\}<\infty .$$

**Proof.**  It can be derived from the relationship established in (25) and the conditions of Assumption 2:

(43) $$∥\sum_{k,B}^{-}∥\leq \left(1+b_{1}\right){\bar{a}}^{2}∥\sum_{k,B}∥+\left(1+b_{1}^{-1}\right){\bar{g}}^{2}∥\sum_{k,B}∥+{\bar{b}}^{2}\bar{q}.$$

Through analysis of Equations (26) and (27), the UB for the estimator gain $K_{B}$ can be computed as:

(44) $$∥K_{k,B}∥\leq \frac{\bar{\theta }\bar{e}\bar{c}}{{\underset{-}{\theta }}^{2}{\underset{-}{e}}^{2}{\underset{-}{c}}^{2}}≜\bar{k}.$$

Denote $\bar{\kappa }≜∥I-K_{k,B}{\bar{L}}_{k,B}{\bar{D}}_{k,B}C_{k,B}∥$, then it can be obtained from (25) that

(45) $$\begin{array}{cc} \sum_{k,B}= & \left(1+b_{2}\right){\bar{\kappa }}^{2}∥\sum_{k,B}^{-}∥+\left(1+b_{2}^{-1}\right)\left(1+b_{3}\right){\bar{k}}^{2}\bar{\mu }+\left(1+b_{2}^{-1}\right)\\ \; & \;\times \left(1+b_{3}^{-1}\right){\bar{k}}^{2}{\bar{\theta }}^{2}{\bar{c}}^{2}∥\sum_{k,B}^{-}∥. \end{array}$$

Combing (43) and (45), it can be obtained that:

(46) $$\begin{array}{cc} ∥\sum_{k,B+1}∥\leq & \left(\left(1+b_{2}\right){\bar{\kappa }}^{2}+\left(1+b_{2}^{-1}\right)\left(1+b_{3}^{-1}\right){\bar{k}}^{2}{\bar{\theta }}^{2}{\bar{c}}^{2}\right)(\left(1+b_{1}\right){\bar{a}}^{2}\\ \; & \;+\left(1+b_{1}^{-1}\right){\bar{g}}^{2})∥\sum_{k,B}∥+(\left(1+b_{2}\right){\bar{\kappa }}^{2}+\left(1+b_{2}^{-1}\right)\left(1+b_{3}^{-1}\right)\\ \; & \;\times {\bar{k}}^{2}{\bar{\theta }}^{2}{\bar{c}}^{2}){\bar{b}}^{2}\bar{q}+\left(1+b_{2}^{-1}\right)\left(1+b_{3}\right){\bar{k}}^{2}\bar{\mu }. \end{array}$$

since

$$\left(\left(1+b_{2}\right){\bar{\kappa }}^{2}+\left(1+b_{2}^{-1}\right)\left(1+b_{3}^{-1}\right){\bar{k}}^{2}{\bar{\theta }}^{2}{\bar{c}}^{2}\right)\left(\left(1+b_{1}\right){\bar{a}}^{2}+\left(1+b_{1}^{-1}\right){\bar{g}}^{2}\right)<1.$$

Through the previous discussion, it can be followed directly from Lemma 3, Theorem 1 and (39) that the norm of $∥\sum_{B+1}∥$ eventually converges. This completes the proof.    □

Building upon these analytical results, the complete JSFE procedure is presented in Algorithm 1.
**Algorithm 1** The designed JSFE algorithm**Input:** initial conditions ${\bar{\xi }}_{0},\;{\hat{\xi }}_{0},\;P_{0},\;\sum_{0}$; process and measurement noise $Q_{B},\;R_{i,B}$; total simulation times *N*.**Output:** state estimation values ${\hat{\xi }}_{B}$ and fault estimate value ${\hat{f}}_{B}$.**step** **1:**Denote B=0;**step** **2:**Calculate prediction ${\hat{\xi }}_{k,B}^{-}$ and the censoring probability ${\bar{\gamma }}_{k,B}$ (12) and (10);**step** **3:**Compute the UB on the local PEC $\sum_{k,B}^{-}$ and local filter gain $K_{k,B}$ according to (25) and (26);**step** **4:**Obtain the UB on the local FEC $\sum_{k,B}^{\min}$ via (27);**step** **5:**Calculate the fusion state estimation ${\hat{\xi }}_{B}$ and fault estimate ${\hat{f}}_{B}$ by (39);**step** **6:**If B≤N, set B=B+1 and return to step 2, else go straight to step 7;**step** **7:**Stop.

**Remark** **2.** *The computational burden of the proposed JSFE algorithm is primarily influenced by the calculation of the local filter gain and the federated fusion step. While the federated fusion rule introduces additional matrix operations, the decentralized structure allows for parallel processing at sensor nodes, which alleviates the real-time constraints on the fusion center. However, for large-scale systems with many sensors, future optimization (e.g., sparse gain computation or event-triggered updates) may be necessary to ensure scalability.*

**Remark** **3.** *For the proposed JSFE method, the key parameters include the sensor noise covariance matrix, the fusion weight factor, and the fault detection threshold. The selection of these parameters should be guided by the characteristics of the sensor network, such as the level of measurement noise and the number of sensors. For instance, higher sensor noise may require larger threshold values for fault detection to minimize false positives.*

## 4. Illustrative Example

**Example** **1.** *Consider the positioning problem of wheeled robots proposed in complex indoor environments [47]. In practical applications, the position information of robots is collected by multiple sensors. Due to the occlusion of the target or the limitation of environmental impacts, the phenomena of MMs and MC may occur. In addition, robots are also prone to actuator failures. Based on the above phenomena, the simulation parameters for* (1) *and* (4) *are chosen as:*

(47) $$\begin{array}{c} {\vec{\xi }}_{B}=\left[\begin{array}{c} {\hat{x}}_{B}\\ {\hat{y}}_{B}\\ {\hat{\theta }}_{B} \end{array}\right],\;\left[\begin{array}{c} \frac{\Delta S_{R}+\Delta S_{L}}{2}\cos\left({\hat{\theta }}_{B}\right)\\ \frac{\Delta S_{R}+\Delta S_{L}}{2}\sin\left({\hat{\theta }}_{B}\right)\\ \frac{\Delta S_{R}-\Delta S_{L}}{b} \end{array}\right],\\ {\vec{A}}_{B}=\left[\begin{array}{ccc} 1 & 0 & 0\\ 0 & 1 & 0\\ 0 & 0 & 1 \end{array}\right],\;{\vec{B}}_{B}=\left[\begin{array}{ccc} 0.1 & 0 & 0\\ 0 & 0.1 & 0\\ 0 & 0 & 0.1 \end{array}\right],\\ {\vec{C}}_{1,B}={\vec{C}}_{2,B}={\vec{C}}_{3,B}=\left[\begin{array}{ccc} 1 & 0 & 0\\ 0 & 1 & 0 \end{array}\right],\;{\vec{F}}_{B}=\left[\begin{array}{cc} 1 & 0\\ 0 & 1 \end{array}\right]. \end{array}$$

*where $\left({\hat{x}}_{B},{\hat{y}}_{B}\right)$ and ${\hat{\theta }}_{B}$ are the position and orientation, respectively. b represents the track width of the two rear drive wheels, V is the current direction of motion for robots, $\Delta S_{R}$ and $\Delta S_{L}$ represent the respective travel distances of the wheels over the specified time interval, and the censoring thresholds are set as $C_{1}=C_{2}=C_{3}={\left[\begin{array}{cc} -15 & -2 \end{array}\right]}^{T}$. The other parameters are selected as ${\bar{D}}_{B}=0.1,{\vec{G}}_{B}=1,{\vec{Q}}_{B}=3I,{\vec{Q}}_{B}^{f}=0.0025I$, $R_{1,B}=R_{2,B}=R_{3,B}=5I,\;b_{1}=0.4$, $b_{2}=0.8,\;b_{3}=0.25$. The initial values are given as follows: ${\vec{\xi }}_{0}={\left[\begin{array}{ccc} 0 & 0 & -135 \end{array}\right]}^{T}$, and the actuator fault is described as:*

$$f_{B+1}=\left\{\begin{array}{cc} 0 & 0\leq B\leq 30,\\ f_{B}+0.05 & \;\mathrm{else}.\; \end{array}\right.$$

**Remark** **4.** *The robot navigation example reflects common challenges in indoor environments such as occlusions, sparse sensor data, and fault-prone actuators. The scenario provides a realistic platform to verify the JSFE algorithm’s anti-disturbance capability under MC and MM phenomena.*

**Remark** **5.** *The actuators of indoor mobile robots may experience slowly varying additive faults due to mechanical wear or environmental disturbance. Examples include motor torque degradation caused by bearing wear or sensor bias drift. Early detection of such faults requires model-based or data-driven approaches, and fault-tolerant performance can be enhanced through adaptive control strategies.*

**Figure 2:**
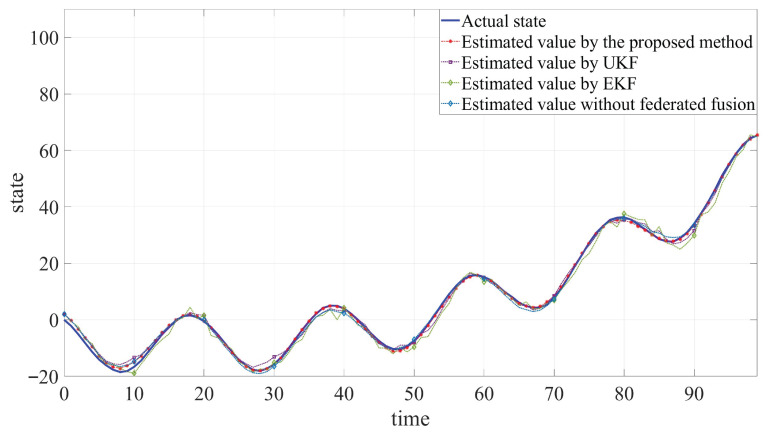
Comparison between estimated values and actual state of $\xi_{B}^{1}$.

**Figure 3:**
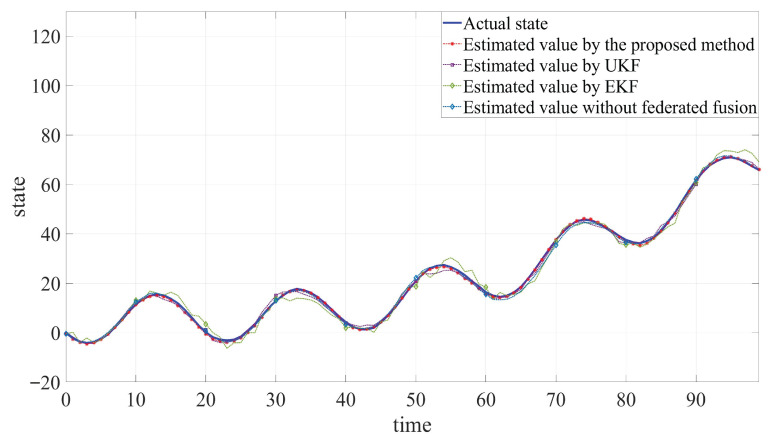
Comparison between estimated values and actual state of $\xi_{B}^{2}$.

**Figure 4:**
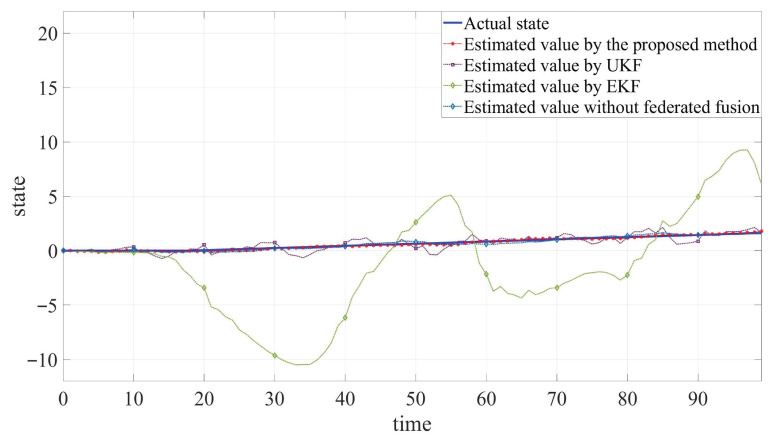
Comparison between estimated values and actual value of fault $f_{B}$.

The developed JSFE algorithm is implemented and validated in MATLAB (R2020a). And it is compared with common filtering methods and the method without fusion center. The simulation results, presented in Figure 2, Figure 3 and Figure 4 and Table 1, demonstrate the algorithm’s effectiveness. Figure 2 and Figure 3 display the actual states $\xi_{B}^{1}$ and $\xi_{B}^{2}$ along with their estimated values by different methods. Figure 4 shows the fault signal and its estimated value of the actuator fault by different methods. Although MMs exhibit certain impacts on estimation performance, the designed JSFE framework demonstrates the capability to restore estimation accuracy within a short period following MMs. Meanwhile, the estimation effect is significantly better than that of other methods.

As shown in Table 1, the RMSE values obtained by the proposed algorithm are consistently lower than those of UKF and EKF. This improvement can be attributed to the Tobit model’s superior ability to handle censored data compared to the conventional filtering methods. Additionally, it can be observed that the RMSE increases when federated fusion is applied, as opposed to using individual filters alone. Although federated fusion may impose a higher computational or communication burden on the system, it results in more accurate state estimation. In practical engineering applications, it is essential to dynamically balance the trade-off between communication load and the number of sensors involved in the fusion process, based on specific system requirements. Simulation outcomes and quantitative analysis verify the algorithm’s capability to simultaneously estimate states and faults under MC, MMs, and actuator fault conditions, validating the anti-disturbance capability of the proposed JSFE method.

**Example** **2.** *This illustrative case employs an oscillator configuration, adopting the parameter set specified in [51]:*

$$\begin{array}{cc} & {\vec{A}}_{B}=\left[\begin{array}{cc} \cos(\omega ) & -\sin(\omega )\\ \sin(\omega ) & \cos(\omega ) \end{array}\right],{\vec{B}}_{B}=\left[\begin{array}{c} 0.1\\ 0.15 \end{array}\right],{\vec{Q}}_{B}=0.0025I,\\ & {\vec{C}}_{1,B}=\left[\begin{array}{cc} 1 & 0 \end{array}\right],{\vec{C}}_{2,B}=\left[\begin{array}{cc} 0 & 1 \end{array}\right],{\vec{F}}_{B}=\left[\begin{array}{cc} 1 & 0\\ 0 & 1 \end{array}\right],\\ & {\vec{R}}_{1,B}={\vec{R}}_{2,B}=0.6I,\omega =0.052\pi ,C_{1}=-4.5,C_{2}=-18. \end{array}$$

*The initial values are given as follows: ${\vec{\xi }}_{0}={\left[\begin{array}{cc} 6 & 1 \end{array}\right]}^{T}$ and the actuator fault is described as:*

$${\vec{f}}_{B+1}=\left\{\begin{array}{cc} 0 & 0\leq B\leq 100,\\ -0.5 & \;\mathrm{else}.\; \end{array}\right.$$

**Remark** **6.** *Oscillator bias is a common yet often overlooked type of systematic fault that directly affects time synchronization, data fusion, and the accuracy of state estimation in sensor systems. It is particularly prone to occur in long-duration operations, harsh environmental conditions, and low-cost hardware platforms. Accounting for and compensating for this bias is of significant engineering relevance, as it enhances the anti-disturbance capability of the overall system. Moreover, biased or unstable oscillators can lead to intermittent MMs and MC during multi-sensor fusion, further complicating the estimation process.*

**Figure 5:**
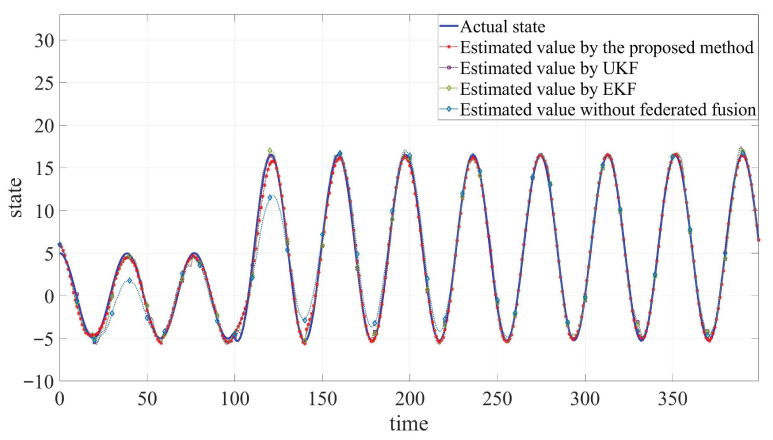
Comparison between estimated values and actual state of $\xi_{B}^{1}$.

**Figure 6:**
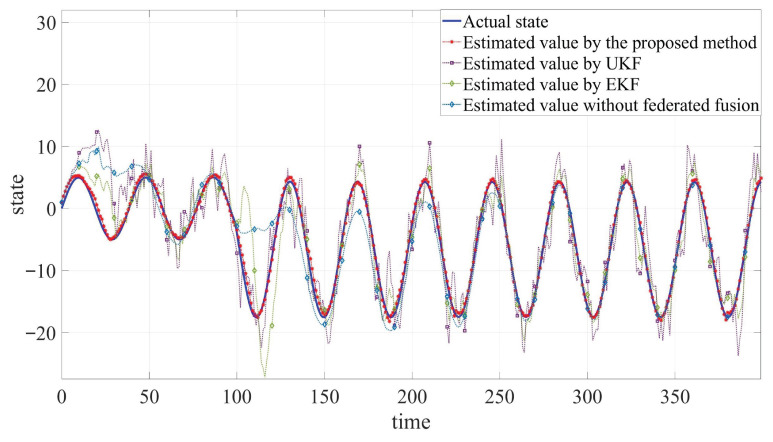
Comparison between estimated values and actual state of $\xi_{B}^{2}$.

**Figure 7:**
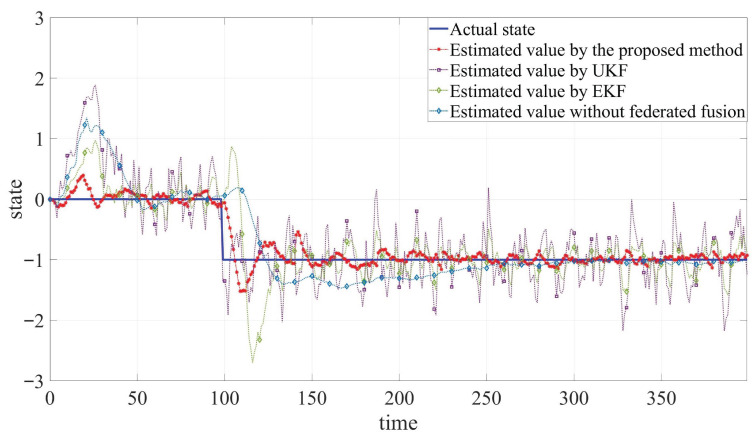
Comparison between estimated values and actual value of fault $f_{B}$.

The remaining parameters are kept consistent with Example 1. The simulation results are shown in Figure 5, Figure 6 and Figure 7 and Table 2.

Even if a step fault occurs, the system can still estimate the fault signal in a short time. As shown in Table 2, for $\xi_{B}^{1}$, where no measurement loss occurs, the proposed method performs slightly better than UKF and EKF. In contrast, for $\xi_{B}^{2}$, which is affected by measurement loss, the proposed method significantly outperforms the others. These results demonstrate that the proposed estimation scheme achieves effective joint tracking of states and faults. The proposed algorithm achieves satisfactory performance. These comprehensive simulation outcomes collectively validate the effectiveness of the proposed filtering approach in real-world applications. It can be seen that even if a step fault occurs, the system can still estimate the fault signal in a short time. These results demonstrate that the proposed estimation scheme achieves effective joint tracking of states and faults. The proposed algorithm achieves satisfactory performance. These comprehensive simulation outcomes collectively validate the effectiveness of the proposed filtering approach in real-world applications.

**Remark** **7.** *For fair comparison, the proposed filter, the EKF, and the UKF are configured with identical process noise covariance ${\vec{Q}}_{B}$, measurement noise covariance ${\vec{R}}_{B}$, and initial state conditions. For the UKF, the scaling parameters are set to $\alpha =10^{-3}$, β=2, and κ=0, consistent with standard recommendations for Gaussian noise assumptions. With a state dimension of n=−5, this yields a scaling factor of λ≈−5 for sigma-point generation. All other tuning parameters were kept the same across methods to ensure an unbiased performance comparison.*

## 5. Conclusions

This paper investigates the JSFE problem of nonlinear systems based on multi-sensor fusion in the event of actuator failure, while considering both MC and MMs. In the estimation process, to address the limitations of a single sensor, federated fusion is used to improve data accuracy and reliability. This paper proposes a new JSFE filter based on recursive thinking, which designs the local estimator gain reasonably through a series of mathematical operations, and based on this, provides a unified filtering fusion framework through the proposed JSFE algorithm. In addition, sufficient conditions are established to guarantee bounded estimation error in the mean square sense. Finally, the effectiveness of the proposed JSFE algorithm is verified through simulation of two engineering examples. While the proposed algorithm demonstrates promising performance in simulation-based validation, future work will explore its robustness under more complex nonlinear dynamics and noisy environments, which are not fully captured in the current study. The proposed framework assumes idealized conditions such as synchronous measurements and reliable communication links. In practical wireless sensor networks, however, data transmission may suffer from delays, packet drops, or asynchronous arrivals. These problems may affect the performance of JSFE algorithms. Future work will focus on enhancing the robustness and anti-disturbance capability of the proposed estimator under these non-ideal conditions, potentially through the incorporation of delay-compensated filtering, adaptive communication protocols, and sensor reliability modeling.

## Figures and Tables

**Table 1:** Estimation performance of Example 1.

Algorithm	RMSE of $\xi_{B}^{1}$	RMSE of $\xi_{B}^{2}$	RMSE of $f_{B}$
The Proposed Method	0.7223	0.4845	0.0859
UKF	1.3463	1.1082	0.4041
EKF	2.0065	2.1673	4.9826
Without federated fusion	1.0503	0.9149	0.1431

**Table 2:** Estimation performance of Example 2.

Algorithm	RMSE of $\xi_{B}^{1}$	RMSE of $\xi_{B}^{2}$	RMSE of $f_{B}$
The Proposed Method	0.4662	0.5468	0.1481
UKF	0.5560	4.3708	0.5132
EKF	0.5269	3.4079	0.4032
Without federated fusion	0.6239	3.8112	0.3961

## Data Availability

The original contributions presented in the study are included in the article, further inquiries can be directed to the corresponding author.
